# Construal level and free will beliefs shape perceptions of actors' proximal and distal intent

**DOI:** 10.3389/fpsyg.2015.00777

**Published:** 2015-06-08

**Authors:** Jason E. Plaks, Jeffrey S. Robinson

**Affiliations:** Department of Psychology, University of TorontoToronto, ON, Canada

**Keywords:** intentionality, moral judgment, psychological distance, free will beliefs

## Abstract

Two components of lay observers' calculus of moral judgment are *proximal intent* (the actor's mind is focused on performing the action) and *distal intent* (the actor's mind is focused on the broader goal). What causes observers to prioritize one form of intent over the other? The authors observed whether construal level (Studies 1–2) and beliefs about free will (Studies 3–4) would influence participants' sensitivity to the actor's proximal vs. distal intent. In four studies, participants read scenarios in which the actor's proximal and distal intent were independently manipulated. In Study 1, when only distal intent was present in the actor's mind, participants rated the psychologically distant actor more responsible than the psychologically near actor. In Study 2, when only distal intent was in the actor's mind, participants with a chronic high level of action identification rated the actor more responsible than did those with a low level of action identification. In both studies, when only proximal intent was in the actor's mind, construal level did not predict judgments of responsibility. In Study 3, when only proximal intent was present in the actor's mind, the more participants believed in free will, the more they rated the actor responsible. When only distal intent was in the actor's mind, free will belief did not influence ratings of responsibility. In Study 4, the same pattern emerged when free will/determinism beliefs were manipulated and the actor performed a positive (life-saving) act. The authors discuss how these results shed new light on the literatures on moral reasoning and psycho-legal theory.

## Introduction

The concept of intentionality holds a central position in both scholars' and laypeople's understanding of moral responsibility (Davidson, 1980; Searle, 1983; Bratman, 1987; Malle and Knobe, 1997). Numerous studies have demonstrated that people generally hold that there is a positive relationship between the degree of intentionality of an act and the actor's degree of moral responsibility (Pizzaro et al., 2003; Reeder, 2009; Guglielmo and Malle, 2011; Laurin and Plaks, 2014).

Considerably less research, however, has investigated exactly how people calculate the degree to which an act is “intentional” (for exceptions, see Shaver, 1985; Malle and Knobe, 1997). This is despite the fact that in many of the thorniest moral and legal dilemmas the actor commits the act with only partial awareness or control (e.g., Hart and Honore, 1959; Duff, 1990). To begin to illustrate varieties of ambiguous intent, consider the following:

J.G. wanted to kill his rich uncle, as he stood to inherit a large sum of money. He decided to kill his uncle by running him down with his car. He began to drive speedily to his uncle's home. As he drove, he thought about killing his uncle. While approaching his uncle's house, J.G. noticed a person crossing into the path of the car. Startled to see a person in the road, J.G tried to press the brake but accidentally pressed the accelerator instead. The car struck the pedestrian, killing the pedestrian instantly. The pedestrian turned out to be his uncle.

In this scenario (adapted from Chisholm, 1966), the means and the end have been decoupled; although J.G. desired his uncle's death, played a role in causing the death, and was in fact thinking about killing his uncle at the time of the death, the death blow occurred outside of J.G.'s control. Philosophers and psychologists have referred to this type of scenario as “causally deviant” (e.g., Davidson, 1980; Mitchell, 1982). In several studies Pizzaro et al. (2003) found that, despite the presence of identical motives and outcomes, participants rated actors in causally deviant scenarios less responsible than actors in scenarios in which the murder proceeded according to plan. This finding suggested that perceivers do not always consider intentionality a binary, “either/or” concept. At times, they are sensitive to gradations of intent and, in turn, moral responsibility.

## Proximal intent and distal intent

Contemporary theories of moral judgment, to the extent that they include an intentionality component, tend to operationalize intentionality in explicitly binary terms; i.e., “intentional” or “unintentional” (e.g., Cushman, 2008; Young and Saxe, 2008; Malle et al., 2014). While agreeing that lay perceivers often use a binary distinction between intentional and unintentional action, Plaks et al. (2009b) proposed that when intentionality is ambiguous, observers turn to a more incremental model. The authors posited that the lay concept of intentional action contains at least two subcomponents: *proximal intent* and *distal intent*. Plaks et al. (2009b) defined proximal intent as the exercise of awareness and control over the physical performance of the critical act (i.e., doing in “on purpose”). In other words, the actor's mind is focused on the immediate aim of task execution. Distal intent is defined as the performance of the act as a means to an end. That is, during the act, the actor's mind is focused on a larger aim, beyond the act itself.

Put differently, proximal intent describes intentionality with respect to the means, whereas distal intent describes intentionality with respect to the end. Although philosophers and legal scholars have made related conceptual distinctions between, for example, “intention” vs. “intention-in-action” (Searle, 1983), “prospective” vs. “concurrent” intention (Brand, 1984), “oblique” vs. “direct” intention (Bentham, 1789/1970), and “bare intention” vs. “intentional action” (Duff, 1990), to our knowledge there have been few attempts to operationalize and measure laypeople's use of these concepts using the tools of contemporary experimental psychology.

### PI and DI may vary independently

For most mundane intentional acts (e.g., picking up a coffee cup), both proximal intent and distal intent operate in tandem; through the conscious application of force the actor accomplishes the goal. However, our central hypothesis is that proximal and distal intent may vary independently. Thus, at the moment of the act, the actor may be more focused on one form of intent than the other. For example, when the actor's mind is firmly on accomplishing a larger goal, but the outcome is reached through coincidence, the act has occurred with distal intent more prominent than proximal intent. (The “J.G.” scenario presented above fits into this category). On the other hand, an act that occurs with the actor focused primarily on the details of execution—as opposed to the larger goal—has occurred with proximal intent more prominent than distal intent. For example, imagine that J.G. (who wants to kill his uncle), while practicing shooting his gun, focuses intently on his firing technique. While killing the uncle is not on J.G.'s mind at that exact moment, a bullet shoots out and happens to hit the uncle. In short, one might represent the presence or absence of proximal intent and distal intent using a 2 (distal intent: present vs. absent) × 2 (proximal intent: present vs. absent) framework.

We wish to note that although in the present studies we operationalized distal and proximal intent dichotomously (i.e., either present or absent), it is more likely that people mentally represent the presence/absence of proximal and distal intent as continua. Thus, it is possible that, for example, when an actor performs an action with proximal intent present, distal intent is never completely absent. As such, our labels for the conditions in the present studies (e.g., Distal Intent Only,” “Proximal Intent Only”) should be interpreted as shorthand for more cumbersome labels like “Proximal Intent more prominent in consciousness than Distal Intent.” Accordingly, we suggest that laypeople's judgments of responsibility are sensitive to cases when each form of intent is *more* vs. *less* present.

Why is it important to distinguish between proximal and distal forms of intent? Designs that independently manipulate the presence or absence of both forms of intent may allow researchers to pinpoint with greater precision how different psychological variables influence perceptions of intent and, in turn, responsibility. For example, some variables may attune perceivers to whether the actor performed the act with awareness and control (proximal intent) more than whether the actor performed the act as a means to an end (distal intent). Other variables may attune participants to distal intent more than to proximal intent. In Studies 1 and 2 we present evidence that (high level vs. low level) action construal is associated with greater emphasis on the actor's distal intent. In Studies 3 and 4 we present evidence that higher belief in free will is associated with greater emphasis on the actor's proximal intent.

### Distinguishing distal intent from related concepts

Distal intent differs from the concept of “desire.” Desire refers to a preference or wish for a particular outcome. People, however, do not always act on their desires. For example, an individual may harbor a sexual desire toward another individual, but never act on it (e.g., Cohen and Rozin, 2001). An intentional act, in contrast, goes beyond thought to goal-directed action (Brand, 1984; Malle, 2004). Thus, we operationalize distal intent as not merely the actor's desire, but the actor's representation that the act *currently being performed* is aimed toward a specific end. In the present studies, desire was kept constant by being present in all conditions (e.g., J.G. wishes his uncle to die). Distal intent, however, was manipulated by being present in the actor's mind in some conditions, but absent in others.

**Figure 1:**
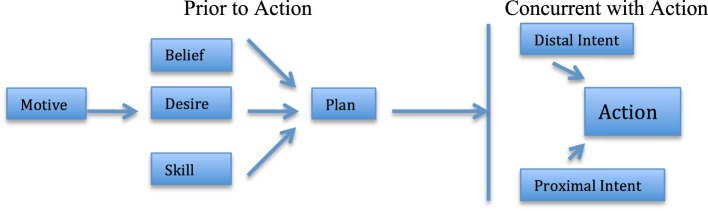
**A model depicting the relationships among proximal intent, distal intent, and other related constructs**.

Distal intent also differs from such legal and philosophical concepts as “motive” or “plan” (Kenny, 1978; Duff, 1990). A motive refers to aspects of the actor's station in life that could plausibly explain why he or she performed the act (e.g., J.G. stands to inherit money from his uncle). A plan refers to the actor's prearranged set of instructions for performing the act. Distal and proximal intent refer to thoughts in the actor's conscious mind *the moment the act is taking place*. Thus, whereas motive and plan refer to preexisting thoughts that may be independent of the act itself, distal intent, by definition, can only be present when the act is occurring. In the present studies, participants read scenarios in which the actor's motive and plan were kept constant across all conditions, while distal and proximal intent were manipulated. In Figure 1, we suggest one possible way to represent the relationships among these various constructs.

One model that, to our knowledge, has come closest to focusing on the actor's in-the-moment thought is Malle and Knobe model (1997), which identified “awareness of the act while the person is performing it” as one of five key components of the folk theory of intentionality. In one study that manipulated aspects of awareness, the authors presented participants with a scenario in which an actor learned a technique to cheat cashiers. The authors manipulated whether the actor “left the store knowing he received too much change” or “left the store unaware that he received too much change.” It is not clear from this manipulation, however, what specifically the actor was thinking about during the act itself—e.g., the details of executing the scam, a broader goal to use the ill-gotten money to buy a new stereo, both, or neither? The present research aims to address these distinctions.

Another literature that has focused on similar issues is research on the “side-effect effect” (Knobe, 2003; Laurent et al., 2015). In addressing the issue of whether the “badness” of an action's outcome influences the degree to which the action is viewed as intentional, researchers have helpfully pointed out that people distinguish between “intentions” (which are associated with overarching goals) and “intentionality” (which is associated with the means to furthering those goals) (Laurent et al., 2015). In the present studies, we extend this literature by holding the outcome constant. We suggest that, independently of the severity of the outcome, lay perceivers generally distinguish between proximal and distal forms of intent.

### Preliminary evidence

Plaks et al. (2009b) presented participants with scenarios in which the actor's proximal and distal intent were independently manipulated. They found that participants rated the actor with both forms of intent in mind most responsible, an actor with one but not the other partially responsible, and an actor with neither form of intent least responsible. Moreover, Plaks et al. (2009b) isolated psychological variables that predict whether perceivers' judgments will be more influenced by the presence or absence of proximal intent or distal intent. One such variable was whether participants were primed with a “psychodynamic” or “cognitive control” model of the human mind. Those who read an article touting evidence that “people's desires inevitably get expressed in behavior, such as ‘Freudian slips”’ were more sensitive to whether the actor had the malevolent goal in mind (i.e., whether distal intent was present or absent) than whether the act was performed on purpose (i.e., whether proximal intent was present or absent). In other words, from the psychodynamic perspective, because desires lead inexorably to their corresponding actions, simply having the malevolent goal in mind is sufficient grounds for punishment. Those primed with an article touting evidence that “people are capable of controlling even their deepest wishes and desires” were more focused on whether proximal intent was present or absent than whether distal intent was present or absent. In other words, from the cognitive control perspective, control over the mechanics of action is the key. Thus, performing the act with awareness and control is sufficient grounds for punishment, even if the actor did not believe at the time that he was committing murder.

Such data provide initial evidence that the Proximal Intent/Distal Intent framework represents an effective tool for isolating different observers' points of emphasis as they determine an actor's level of responsibility. In the present studies, participants read scenarios in which we independently manipulated whether distal intent or proximal intent was more prominent in the actor's mind. In Studies 1 and 2, we examined one variable that was hypothesized to affect sensitivity to distal intent: high- vs. low-level construal of action. In Studies 3 and 4, we examined a second variable that was hypothesized to affect sensitivity to proximal intent: belief in free will.

### Why construal level and free will beliefs?

We selected these particular variables for two reasons. First, although both variables have been linked to general increases or decreases in punitiveness toward a wrongdoer (e.g., construal level: Eyal et al., 2008; Gong and Medin, 2012; free will beliefs: Clark et al., 2014; Shariff et al., 2014), neither variable, to our knowledge, has been applied to the perception of the actor's intent. Second, for both variables, the evidence regarding moral judgment is not entirely clear. For example, whereas Eyal et al. (2008) found evidence for a link between high-level construal and higher punishment, Gong and Medin (2012) subsequently presented evidence for the opposite: a link between low-level construal and higher punishment. In addition, whereas several studies have linked higher free will beliefs with higher punishment (e.g., Clark et al., 2014), others have questioned the manner in which free will beliefs have been assessed (e.g., Nadelhoffer et al., 2014).

We suggest that distinguishing between proximal and distal intent may help to advance both literatures by highlighting cases when construal level and free will beliefs do and do not affect participants' judgments. To foreshadow the results, in Studies 1–2 we report evidence that higher construal leads to more extreme moral judgment only when distal intent is more prominent in the actor's mind than proximal intent. In Studies 3–4 we report evidence that higher belief in free will predicts more extreme judgment in all cases *except* when distal intent is more prominent in the actor's mind than proximal intent.

## Study 1

The same action may be mentally represented at different levels of abstraction (e.g., Vallacher and Wegner, 1987; Maass et al., 1989). For example, “following lines of print,” “reading,” and “acquiring knowledge” might all be used to describe the same act. What determines whether people will select a higher, more abstract construal or a lower, more concrete construal?

One answer appears to be psychological distance. Numerous studies indicate that higher psychological distance elicits higher level construals (e.g., Bar-Anan et al., 2006; for a review, see Trope and Liberman, 2010). Psychological distance has been operationalized in a variety of ways, including physical distance (e.g., Maglio et al., 2013), temporal distance (e.g., Nussbaum et al., 2003), and social power (Smith and Trope, 2006). In the present study, we manipulated the physical distance of the actor from the Canadian participants: half of the participants were led to believe that the event occurred in Russia.

We hypothesized that actors judging the distant target would be more sensitive to the presence or absence of distal intent. This is because a higher level construal goes beyond the physical motion to include the actor's aim or purpose (Kozak et al., 2006). Thus, we expected that when the actor had the malevolent distal aim in mind but performed the act by accident, participants in the distant condition would judge the actor more harshly than would those in the near condition. Proximity, in contrast, encourages a more concrete representation that focuses on means, rather than outcomes (e.g., Liberman and Trope, 1998). Thus, we expected that when the actor performed the physical act with awareness and control but did not have the malevolent distal intent in mind, participants in the near condition would judge the actor more harshly than would those in the distant condition. Statistically speaking, the hypothesis was for two main effects (Distal Intent: present vs. absent; Proximal Intent: present vs. absent) and two two-way interactions (Distal Intent × Distance; Proximal Intent × Distance).

## Method

### Participants

A total of 149 (77 males) undergraduates at the University of Toronto participated in one session in a lecture setting. (Ethnic breakdown: 44% White, 29% East Asian, 11% South Asian, all remaining identities <10%.) Sample size was determined by the number of students in the class. All participants provided informed consent. This study and all subsequent studies received ethics approval from the University of Toronto Research Ethics Board (Social Sciences, Humanities, and Education REB).

### Procedure

Participants were randomly assigned to receive one of eight booklets of printed materials. In all scenarios, participants were introduced to “Alex” and “Linda” with accompanying photos. In all scenarios Alex's desire (to kill Linda) and plan (to drown her in a lake) were kept constant while intent was manipulated. In the Both Present condition, Alex had the distal intent to kill Linda in mind as he carefully and purposefully executed his plan to tie her up and drown her in the lake. In the Distal Intent Only condition, Alex had the distal intent (killing Linda) in mind at the moment when the outcome occurred in a causally deviant manner (Linda died while attempting to escape). In the Proximal Intent Only condition, Alex did not have the intent to kill Linda in mind at the moment when he purposefully performed the action (pulling a rope) that ended up killing Linda. In the Both Absent condition, he had neither the distal intent in mind, nor did he perform the death blow on purpose (i.e., proximal intent was not in mind).

To summarize, distal intent was manipulated by whether Alex's mind at the moment of the death blow was vs. was not focused on killing Linda. Proximal Intent was manipulated by whether the death blow occurred under Alex's control vs. not under Alex's control. All other content was held constant. (See Supplementary Material).

To manipulate distance, half of the participants read that the event occurred in Russia with characters named Igor and Svetlana. The same photos were used in both the near and far conditions. Note that the use of Russian vs. Canadian characters manipulates not only physical distance, but social distance (Liviatan et al., 2008). Social distance manipulations have been found to yield effects parallel to those of other forms of distance, including physical and temporal distance (Maglio et al., 2013).

After reading one of the eight scenarios, participants provided ratings (on 0–6 scales) on four moral judgment items: “How much moral responsibility does Alex have for what happened to Linda?,” “How intentional was Alex's action?,” “How much blame should go to Alex for what happened to Linda?,” “How negatively should Alex by judged?” We included a range of intentionality, blame, and punishment items to assess whether participants would exhibit any divergences between ascriptions of intentionality and punishment (e.g., Cushman et al., 2012).

## Results and discussion

Because the moral judgment items were highly correlated (Cronbach's α = 0.94), they were combined into a moral judgment index. Participants' scores on the moral judgment index were submitted to a 2 (distance: near vs. far) × 2 (distal intent: present vs. absent) × 2 (proximal intent: present vs. absent) between-subjects ANOVA.

### Overall effects

This analysis revealed the predicted main effects for distal intent, *F*_(1, 141)_ = 47.80, *p* < 0.001, and proximal intent, *F*_(1, 141)_ = 12.51, *p* = 0.001. Not surprisingly, participants rated the actor with both forms of intent present (*M* = 5.69, *SD* = 0.81) substantially more responsible than the actor with both absent (*M* = 3.67, *SD* = 1.43), *F*_(1, 141)_ = 52.12, *p* < 0.001, *d* = 1.80. Participants also rated the actor with only distal intent in mind (*M* = 5.07, *SD* = 1.27) less responsible than the actor with both present, *F*_(1, 141)_ = 4.76, *p* < 0.05, *d* = 0.59, but more responsible than the actor with both absent, *F*_(1, 141)_ = 23.91, *p* < 0.001, *d* = 1.04. Similarly, participants rated the actor with only proximal intent in mind (*M* = 4.34, *SD* = 1.26) less responsible than the actor with both present, *F*_(1, 141)_ = 23.13, *p* < 0.001, *d* = 1.30, and more responsible than the actor with both absent, *F*_(1, 141)_ = 5.79, *p* < 0.05, *d* = 0.50.

### Effects involving distance

Participants in the near vs. far conditions did not differ in the overall harshness of their judgment of Alex, *F*_(1, 141)_ = 1.03, *p* = 0.31. However, the predicted distal intent x distance interaction, *F*_(1, 141)_ = 6.97, *p* < 0.01, indicated that participants in the two distance conditions were differentially influenced by the presence or absence of distal intent. In addition, the predicted proximal intent x distance interaction, *F*_(1, 141)_ = 5.82, *p* < 0.05, suggested that participants in the two distance conditions were differentially influenced by the presence or absence of proximal intent.

Investigating the distal intent × distance interaction first, participants in the far condition rated the actor with distal intent present (i.e., the mean of the Both Present and Distal Intent Only conditions) (*M* = 5.70, *SD* = 0.74) more responsible than the actor with distal intent absent (i.e., the mean of the Both Absent and Proximal Intent Only conditions) (*M* = 3.87, *SD* = 1.28), *F*_(1, 145)_ = 45.36, *p* < 0.001, *d* = 1.81. For near condition participants, this difference (*M*_present_ = 5.02 vs. *M*_absent_ = 4.19) was only of one-third the magnitude *F*_(1, 145)_ = 28.94, *p* < 0.001, *d* = 0.59. In other words, although everyone judged the target more responsible when distal intent was present than when it was absent, this difference was greater for participants who were far from the actor.

Turning next to the proximal intent × distance interaction, participants in the near condition rated the actor more responsible with proximal intent present (i.e., the mean of the Both Present and Proximal Intent Only conditions) (*M* = 5.14, *SD* = 1.23) than absent (i.e., the mean of the Both Absent and Distal Intent Only conditions) (*M* = 4.00, *SD* = 1.47), *F*_(1, 145)_ = 11.75, *p* < 0.001, *d* = 0.85. For participants in the far condition, this difference (*M*_present_ = 4.91, *SD* = 1.27 vs. *M*_absent_ = 4.67, *SD* = 1.51) was not significant, *F*_(1, 145)_ = 0.59 *p* = 0.44. In other words, only near participants judged the target more responsible when proximal intent was present than when it was absent.

In a more direct test of our hypotheses, we compared the two distance groups within each scenario condition. Means for the moral sanction index are depicted in Table 1. (Means for each separate moral judgment question are presented in Supplementary Material).

**Table 1:** **Study 1**.

	**Distal Intent**	
**Proximal Intent**	**Present**	**Absent**
**DISTANT CONDITION**		
Present	5.72 (0.78)	4.06 (1.14)
Absent	5.68 (0.71)	3.66 (1.42)
**NEAR CONDITION**		
Present	5.65 (0.87)	4.67 (1.34)
Absent	4.34 (1.42)	3.68 (1.49)

*Moral sanction of the actor (0–6 scale) as a function of psychological distance and the presence or absence of proximal intent and distal intent. (standard deviations in parentheses)*.

In the Distal Intent Only condition, simple effects tests revealed that participants in the far condition (*M* = 5.68, *SD* = 0.71) rated the actor more responsible than did those in the near condition (*M* = 4.34, *SD* = 1.43), *F*_(1, 148)_ = 11.39, *p* = 0.001, *d* = 1.26. In the Proximal Intent Only condition, the effect trended in the opposite direction (*M*_near_ = 4.67, *SD* = 1.34 vs. *M*_far_ = 4.06, *SD* = 1.14), *F*_(1, 148)_ = 2.61, *p* = 0.09, *d* = 0.49. A significant 2 (distance: near vs. far) × 2 (scenario: Proximal Intent Only vs. Distal Intent Only) interaction, *F*_(1, 74)_ = 12.64, *p* = 0.001, confirmed that participants in the near vs. far conditions differed in their view of which single type of intent (proximal vs. distal) carried more weight.

Note that participants in the near and far conditions rated the actor equally responsible in the condition that was clearly “intentional” (Both Present: *M*_near_ = 5.65, *SD* = 0.87 vs. *M*_far_ = 5.72, *SD* = 0.78). The same was true in the condition that was clearly “unintentional” (Both Absent: *M*_near_ = 3.68, *SD* = 1.42 vs. *M*_far_ = 3.66, *SD* = 1.49). In other words, if intentionality had been operationalized in a binary fashion, the data might suggest that psychological distance has no effect on moral judgment. However, with intentionality operationalized in a 2 × 2 fashion, it was possible to pinpoint a specific case when psychological distance yields a clear effect.

To summarize, when the actor did not have the malevolent goal in mind but did produce the death blow on purpose, participants who were near to the actor assigned more responsibility than did participants who were far from the actor. If anything, psychological distance had the reverse effect when the actor had the malevolent goal in mind but did not produce the death blow on purpose. We suggest that this occurred because higher level representations generally include information about the actor's broader aim (Vallacher and Wegner, 1987). Lower level representations focus more on concrete aspects of the action itself. Thus, broadening observers' representation of the action highlights the question of whether the actor had the malevolent underlying aim in mind as he performed the act. In contrast, narrowing observers' representation raises the salience of intentionality related to the physical application of force: i.e., whether the actor performed the physical act with awareness and control. In Study 2 we examined this idea in a different way, using an individual differences approach.

## Study 2

In Study 2 we made three additional changes. First, rather than using scenarios in which the actor causes the death of another person, we used scenarios in which the actor causes a positive outcome. Do perceivers apply an analogous framework to their judgments of meritorious acts? How do people understand an actor who had a meritorious distal intent in mind but achieved the outcome through serendipity or clumsiness? How do people understand an actor who performed an act in a controlled that turned out to be unexpectedly positive? Put differently, are there asymmetries in the judgment of negative vs. positive actions (e.g., Wiltermuth et al., 2010) or do differences in the abstract vs. concrete mental representation of action affect perceptions of proximal and distal intent equivalently for positive acts and negative acts?

Second, people make intentionality judgments in mundane situations that are morally neutral. Do people extend the PIDI framework to such acts? If so, this would suggest that people consider the independent input of proximal and distal intent when evaluating all actions, not just morally-charged actions. If not, it would suggest that people only apply the PIDI framework to morally-charged actions. To examine this question, in the present study the actor did not kill anyone. Instead, she kicked a soccer ball into a goal.

Third, we examined abstract vs. concrete representation of behavior via individual differences in Action Identification (Vallacher and Wegner, 1987). According to this theory, people may label their own actions (Vallacher and Wegner, 1989) or the actions of others (e.g., Kozak et al., 2006) at a high level or a low level. For example, the same act may be represented as “killing a person” or “pulling a trigger.” Lower level representations focus primarily on procedural or action details with little information regarding why the action was made. Higher level representations include information about “the purpose of the act, its effects, and the particular situation” (Vallacher and Wegner, 1989).

Kozak et al. (2006) documented a general association between higher levels of action identification and higher attributions of intentionality to an actor (especially when the actor was likeable). These researchers did not, however, manipulate the degree to which the actor's act was intentional. We suggest that because higher level action representations take into account the actor's larger purpose, they should be associated with sensitivity to the actor's distal intent. Because lower level representations are focused more on concrete action details, they should be associated with sensitivity to proximal intent. Thus, we hypothesized that observers who chronically tend to make high level identifications would be more influenced than observers who tend to make low level identifications by the relative presence or absence of distal intent in the actor's mind. In contrast, we hypothesized that low level observers would be more influenced than high level observers by the relative presence or absence of proximal intent.

Numerous studies have indicated that people vary reliably in how they identify actions (e.g., Vallacher and Wegner, 1989). To measure individual differences in the tendency to identify actions as a high or low level, we used the 25-item Behavior Identification Form (BIF), which possesses strong psychometric properties (Vallacher and Wegner, 1989).

## Method

### Participants

A total of 468 (255 females) American participants (mean age = 36.14, *SD* = 12.52, minimum = 18, maximum = 76) participated online via Amazon Mechanical Turk in exchange for $0.50 compensation. (Ethnic breakdown: 69% White, 3% Black, 5% Latino, 8% East Asian, 3% South Asian, 1% Middle Eastern, remainder “other.”) Sample size was determined via power analyses (with G^*^Power) using effect sizes reported in previous studies. All participants provided informed consent.

### Procedure

Participants were randomly assigned to read one of four scenarios. In all scenarios participants read that “Jane's soccer team is tied with its arch-rival with 1 min left in the championship game. Jane desperately wants her team to score a goal and win the championship. Jane has the ball at her feet near the opponent's goal.” In the Both Present scenario, Jane aims for the corner of the goal and with precision and control kicks the ball into the net. In the Distal Intent Only condition, she aims for the corner of the goal but the ball veers off course, bounces off a defender, and goes into the net for a goal. In the Proximal Intent Only condition, she aims to pass the ball to her teammate, accurately kicks the ball toward her teammate, but neither the teammate nor the goalkeeper are expecting the pass, and the ball goes past them both into the net for a goal. In the Both Absent condition, Jane aims to pass the ball to her teammate, but the ball veers off course, bounces off a defender, and goes into the net for a goal. Thus, the proximal intent manipulation was whether or not Jane kicked the ball in a controlled, accurate manner toward her target. The distal intent manipulation was whether Jane was aiming for the goal or aiming for her teammate. (For the full text, see Supplementary Material).

After reading their assigned scenario, participants rated Jane on 0–6 scales on items used in Study 1 that were re-worded in terms of a positive outcome: (1) “How intentional was Jane's action?” (2) “How responsible was Jane for the goal?” (3) “How positively should Jane be viewed?” and (4) “How much praise does Jane deserve?”

Next, in what was described as a second, unrelated study, participants completed a series of demographic measures and unrelated questionnaires^1^, among which was the Behavioral Identification Form. On the Behavioral Identification Form (BIF), participants are presented with 25 behaviors. For each behavior, participants must rate the degree to which each act presented at a middle level (e.g., “reading”) is best described at a higher level (“gaining knowledge”) and a lower level (“following lines of print”). Ratings on all items are summed to create an overall BIF score, with higher scores indicating a greater tendency to make high level identifications.

## Results and discussion

### Overall effects

Two participants did not complete any of the moral judgment items, leaving a total sample of 466. Because the moral judgment items were highly correlated (Cronbach's α = 0.71), they were averaged to form an index. Participants' scores on the index were submitted to a regression analysis with Distal Intent, Proximal Intent, BIF score, the two-way interaction terms, and the three-way interaction term entered as predictors. All variables were centered.

As in Study 1, this analysis revealed the predicted main effects for distal intent, β = 0.59, *t*_(459)_ = 9.65, *p* < 0.001, and proximal intent, β = 0.38, *t*_(459)_ = 9.41, *p* < 0.001. There was not a significant overall effect of BIF score, β = 0.06, *p* = 0.15, indicating that high level observers and low level observers did not differ in their overall allocation of praise to Jane.

### Effects involving BIF score (chronic level of action identification)

The analysis also revealed a marginally significant BIF × distal intent interaction, β = 0.07, *t*_(459)_ = 1.70, *p* = 0.09 and significant BIF × distal intent × proximal intent interaction, β = 0.07, *t*_(459)_ = 1.92, *p* = 0.05. The BIF x proximal intent interaction did not reach significance, *p* = 0.19.

Probing the distal intent × BIF relationship, further analyses revealed that high level identifiers (BIF score > +1 *SD* from the mean) rated the actor with distal intent present more responsible than the actor with distal intent absent, β = 0.33, *t*_(463)_ = 5.33, *p* < 0.001, whereas for low level identifiers (BIF score < − 1 *SD* from the mean), this difference though still significant, was smaller, β = 0.22, *t*_(463)_ = 3.55, *p* < 0.001. In other words, although everyone judged the target more responsible when distal intent was present than when it was absent, this difference was, if anything, greater for high level identifiers.

As in Study 1, we sought a more direct test of our hypothesis by examining the effect of chronic level of action identification on participants' judgment within each scenario condition. Means are presented in Table 2.

**Table 2:** **Study 2**.

	**Distal Intent**	
**Proximal Intent**	**Present**	**Absent**
**HIGH LEVEL ACTION IDENTIFIERS**		
Present	5.18 (0.56)	4.04 (0.15)
Absent	3.96 (0.49)	3.62 (0.07)
**LOW LEVEL ACTION IDENTIFIERS**		
Present	4.92 (0.14)	3.95 (0.09)
Absent	3.57 (0.54)	3.55 (0.02)

*Moral praise of the actor (0–6 scale) as a function of chronic action identification and the presence or absence of proximal intent and distal intent. Values represent point estimates from the regression. “High” and “Low” Level Action Identifiers represent greater than +1 and less than −1 standard deviation from mean of the BIF scale. Standard deviations are in parentheses*.

Chronic level of action identification did not influence ratings in the Both Absent, Both Present, or Proximal Intent Only conditions, all β s < 0.06, *t*s > 0.40. However, in the Distal Intent Only condition, the higher the participant's level of action identification, the more praise he or she allocated to Jane, β = 0.29, *t*_(115)_ = 3.21, *p* = 0.002. Thus, when the actor's goal (kicking the ball into the goal) was in her mind, but the outcome occurred by accident (it flew wildly off a defender but into the goal), participants who generally view acts at a higher level gave the actor more credit than did those who generally view acts at a lower level. This difference replicates a similar comparison in Study 1.

We did not, however, replicate Study 1's finding for the Proximal Intent Only condition (lower level construers assign more responsibility than higher level construers). This may be for a number of reasons. The two studies differed in (a) how distance/abstraction was operationalized, (b) the valence of the event (negative vs. positive), (c) the moral significance of the event (highly charged vs. mundane). Future studies should systematically vary each of these variables individually to isolate the cause for the non-replication. An additional possibility is that psychological distance and action identification generally influence perceptions of distal intent more than they influence perceptions of proximal intent. This would be consistent with previous findings involving temporal distance (Plaks et al., 2009b, Study 2).

To summarize, the results of Study 2 suggest three conclusions. First, distal intent generally appears to be represented at a higher level than proximal intent. This fits with the definition of high level identification as sensitive to the act's larger purpose. Second, observers who chronically viewed acts at a high level were more impressed by the presence or absence of distal intent than were observers who chronically viewed acts at a low level. Third, it appears that people do not only apply proximal and distal intent to understand negative or morally-relevant acts; Study 2 participants applied a similar analysis when assessing the intentionality of actions that were positive and did not possess obvious moral implications.

## Study 3

In Studies 3 and 4 we turned to the question of how observers' beliefs might influence the weight they place on proximal and distal intent. This approach builds on the existing literature on the role of *a priori* beliefs, or “implicit theories,” in moral cognition (Miller et al., 2007) and social cognition more generally (e.g., Molden et al., 2006; Plaks et al., 2012; for a review see, Plaks et al., 2009a).

One logical place to start is with beliefs regarding free will/determinism. The assumption that people possess a large degree of freedom over their thought and action lies at the heart of most legal systems (Hart and Honore, 1959; Duff, 1990), which generally hold that it is only fair to punish a wrongdoer if he or she could have done otherwise. Indeed, recent evidence points to a tight (and bi-directional) relationship in laypeople's minds between belief in free will and punitiveness (Clark et al., 2014; Shariff et al., 2014).

The assumption of free will appears to resonate with popular views; according to one multinational survey 70% of respondents believed that their fate is in their own hands (Jowell et al., 1998). Yet 70% is far from unanimous; studies that have measured belief in free will using self-report measures have found a fair amount of variability (Vohs and Schooler, 2008; Paulhus and Carey, 2011). Moreover, it is not always clear what laypeople mean by “free will”: Does it refer primarily to freedom to *choose* one's actions, freedom to *execute* one's actions, or both?

In Study 3, the primary predictor variable was individual difference variation in belief in free will. How might belief in free will relate to sensitivity to proximal or distal intent? One possibility is that greater belief in free will lead people to focus more on the actor's ultimate aim (distal intent). According to this perspective, because people are capable of selecting their course of action, the presence or absence of malevolent (or benevolent) distal intent is of primary importance in moral judgment. Thus, Hypothesis #1 was that greater belief in free will would lead to greater sensitivity to the presence or absence of distal intent.

An alternative possibility is that people generally understand free will not in terms of selecting one's course of action, but in terms of the exercise of control over execution of the action. According to this perspective, a given desire (e.g., a sexual desire) may enter one's head from ambient stimuli in the environment (e.g., an attractive person walking by), but people have the power to control, re-channel, or prevent the corresponding action (e.g., a sexual advance). This perspective locates free will primarily in terms of self-control—i.e., liberating action from the clutches of desire. This emphasis on action control suggests a greater emphasis on proximal intent. Thus, Hypothesis #2 was that greater belief in free will would lead to greater sensitivity to the presence or absence of proximal intent.

The design of the experiment allowed us to test the full complement of hypotheses. Hypothesis #3 was that greater belief in free will would lead to greater sensitivity to both proximal and distal intent. Hypothesis #4 was the null hypothesis (i.e., belief in free will would have no effect). To test which of these hypotheses was most consistent with the data, we measured belief in free will as an individual difference variable and presented scenarios that manipulated the presence or absence of proximal and distal intent.

## Method

### Participants

A total of 165 undergraduates at a Canadian university (73% female, mean age = 20.47, *SD* = 2.79) participated in one session as part of a lecture class. (Ethnic breakdown: 23% East Asian, 16% White, 12% South Asian, all remaining ethnicities <3%). Sample size was determined by the number of students in the class. All participants provided informed consent.

### Procedure

Participants were randomly assigned to read one of four booklets of materials. We adapted scenarios presented by Pizzaro et al. (2003). In the Both Present condition, an actor (“Barbara”) had the goal of killing her husband (“John”) in mind as she successfully executed her plan to poison him during dinner at a restaurant. In the Distal Intent Only condition, Barbara had the goal (killing John) in mind at the moment when the outcome occurred in a causally deviant manner (John died not because of the poison but because of an allergy to another dish). In the Proximal Intent Only condition, she did not have the goal of killing John in mind at the moment when she purposefully performed the action (pouring the poison) that killed John. In the Both Absent condition, she had neither the goal in mind, nor performed the death blow on purpose. In other words, the proximal intent manipulation was whether the death blow was performed under Barbara's control vs. outside of Barbara's control. The distal intent manipulation was whether Barbara's thoughts at the critical moment were on killing John vs. a different topic. For the complete scenarios, see Supplementary Material.

After reading the scenario, participants indicated their rating (on 0–6 scales) of the actor on the moral judgment items used in Study 1. In addition, we included new items intended to assess further aspects of moral judgment. These included items assessing punishment (“To what extent should Barbara be punished for her action?”), justice (“To what extent does Barbara now have ‘bad karma’ as a result of what occurred?” “To what extent is Barbara likely to get the punishment she deserves?”), wrongness (“To what extent was what Barbara did fundamentally wrong?”), the actor's view of the outcome (“How pleased is Barbara about what happened?”), and the actor's moral character (“To what extent is Barbara a bad person?”).

Finally, participants completed a series of questionnaires that were unrelated to the present study^2^. Embedded within those measures was a validated measure of belief in free will/determinism (FAD; Paulhus and Carey, 2011). This measure contains subscales that distinguish among belief in free will, belief in two types of determinism (scientific, fatalistic), and belief in randomness/unpredictability. The free will subscale contains items such as “People have complete free will” (α = 0.62). The scientific determinism scale contains items such as “Psychologists and psychiatrists will eventually figure out all of human behavior” (α = 0.58). The fatalistic determinism scale contains items such as “I believe the future has already been determined by fate” (α = 0.77). The unpredictability scale contains items such as, “People's futures cannot be predicted (α = 0.66).” Participants indicated their level of agreement with these items on 1–5 scales (1 = strongly disagree… 5 = strongly agree).

## Results and discussion

### Overall effects

Two participants did not complete the moral judgment items, five did not complete the free will/determinism measure, and three completed neither, leaving a total sample of 155. Because all moral judgment items were highly correlated (Cronbach's α = 0.82), we combined them into a moral judgment index. We conducted regression analyses for each of the free will/determinism questionnaire subscales with Distal Intent, Proximal Intent, free will/determinism score, all two-way interaction terms, and the three-way interaction term entered as predictors. All variables were centered.

Replicating the previous studies, the omnibus analysis revealed main effects for distal intent, β = 0.20, *t*_(148)_ = 3.41, *p* < 0.001, and proximal intent, β = 0.37, *t*_(148)_ = 6.56, *p* < 0.001. This analysis also indicated a significant main effect for free will beliefs, β = 0.18, *t*_(148)_ = 3.12, *p* < 0.01, replicating previous demonstrations of an association between free will belief and harsher punishment (Clark et al., 2014; Shariff et al., 2014).

### Effects involving belief in free will

The analysis also revealed a near significant free will belief × proximal intent interaction, β = 0.11, *t*_(148)_ = 1.93, *p* = 0.056. For the test of the free will belief × distal intent interaction, the values were: β = −0.87, *t*_(148)_ = −1.53, *p* = 0.14.

We probed the relationship between belief in free will and sensitivity to proximal intent with further analyses. High free will believers (FAD score > +1 *SD* from the mean) rated the actor with proximal intent present more responsible than the actor with proximal intent absent, β = 0.55, *t*_(152)_ = 5.83, *p* < 0.001. For low free will believers (FAD score < −1 *SD* from the mean) this difference was significantly smaller, β = 0.16, *t*_(152)_ = 1.73, *p* = 0.09. In other words, high believers in free will were more sensitive than low believers to the presence or absence of proximal intent. Analogous analyses revealed that high and low free will believers did not differ significantly in their sensitivity to the presence or absence of distal intent, *p*s > 0.10.

As in the previous studies, the most direct test of our hypotheses was to examine the effect of the predictor variable within each scenario condition. In the Distal Intent Only condition, participants' judgments were not influenced by free will beliefs, β = −0.09, *t*_(47)_ = −0.64, *p* = 0.53. However, in the Proximal Intent Only condition, the higher the participant's free will belief, the more condemnation he or she assigned to the actor, β = 0.52, *t*_(34)_ = 3.48, *p* = 0.001 ^3^. Means are presented in Table 3.

**Table 3:** **Study 3**.

	**Distal Intent**	
**Proximal Intent**	**Present**	**Absent**
**HIGH FREE WILL BELIEVERS**		
Present	5.45 (0.31)	3.86 (0.55)
Absent	3.19 (1.12)	4.13 (0.87)
**LOW FREE WILL BELIEVERS**		
Present	5.02 (0.66)	3.25 (0.54)
Absent	3.26 (0.76)	3.82 (0.74)

*Moral sanction of the actor (0–6 scale) as a function of free will beliefs and the presence or absence of proximal intent and distal intent. Values represent point estimates from the regression. “High” vs. “Low “Free Will Believers represent greater than +1 and less than −1 standard deviation from the mean on the FWD scale. Standard deviations are in parentheses*.

In other words, when the outcome occurred through a conscious application of force, participants who generally believed in free will were more punitive—even if the actor's ulterior goal was not in her mind. In addition, higher free will beliefs predicted higher condemnation in the both present condition, β = 0.55, *p* < 0.001 and the both absent condition, β = 0.32, *p* = 0.05. This is consistent with the documented association between free will beliefs and greater punishment (Shariff et al., 2014. Clark et al., 2014 demonstrate the reverse causal direction: greater motivation to punish precipitates greater belief in free will). A boundary condition to this rule appears to be when the actor has distal intent in mind but not proximal intent. In that case, free will beliefs no longer predicted punitiveness.

Overall, this pattern is most consistent with Hypothesis #2. What explains this pattern? These data suggest that laypeople spontaneously tend to define free will in terms of control over behavior (as opposed to control over thought). That is, while people may acknowledge that immoral thoughts often enter the mind outside of one's control, they believe that individuals have the power to control whether these thoughts become expressed in action. Thus, a higher belief in this form of free will implies a higher belief that the actor could have acted otherwise. Philosophers, psychologists, and legal scholars have held that such a belief in the *plausibility of the counterfactual* is necessary for judgments of moral responsibility (e.g., Duff, 1990; Zeki and Goodenough, 2004).

These data parallel previous data in which the experimenters primed a “cognitive control” lay theory (Plaks et al., 2009b, Study 3). In that study, participants who read a passage indicating that people have the power to control their actions were similarly more sensitive to the presence or absence of proximal intent than they were to the presence or absence distal intent.

### Belief in scientific determinism

We conducted analogous regression analyses with Distal Intent, Proximal Intent, scientific determinism score, all two-way interaction terms, and the three-way interaction term entered as predictors. All variables were centered. This analysis revealed the usual main effects of distal intent and proximal intent, both β s > 0.24, both *p*s < 0.001, but no significant effects involving belief in scientific determinism, all β s < 0.07, all *p*s > 0.22.

### Belief in fatalistic determinism

We conducted analogous regression analyses with Distal Intent, Proximal Intent, fatalistic determinism score, all two-way interaction terms, and the three-way interaction term entered as predictors. This analysis revealed main effects of distal intent and proximal intent, both β s > 0.25, both *p*s < 0.001, and a main effect of fatalistic determinism, β = 0.11, *p* < 0.05 (indicating that higher fatalistic determinism generally predicted harsher condemnation). However, neither the fatalistic determinism × distal intent interaction (β = −0.03, *p* = 0.56) nor the fatalistic determinism × proximal intent interaction (β = −0.27, *p* = 0.63) approached significance. Thus, sensitivity to proximal or distal intent did not vary as a function of belief in fatalistic determinism.

### Belief in unpredictability

We conducted analogous regression analyses with Distal Intent, Proximal Intent, unpredictability belief score, all two-way interaction terms, and the three-way interaction term entered as predictors. This analysis again revealed main effects of distal intent and proximal intent, both β s > 0.24, both *p*s < 0.001, and a main effect of unpredictability belief, β = 0.13, *p* < 0.05. However, neither the unpredictability belief x distal intent interaction (β = −0.08, *p* = 0.12) nor the unpredictability belief × proximal intent interaction (β = −0.27, *p* = 0.63) were significant. Thus, sensitivity to proximal or distal intent did not appear to vary as a function of belief in randomness.

To summarize, we found that free will beliefs influenced sensitivity to proximal intent, but not distal intent. Belief in determinism (either type) and belief in randomness did not influence sensitivity to proximal intent or distal intent. Future studies should investigate the relationship between free will beliefs and sensitivity to proximal intent by uncovering mediating mechanisms. For example, is the effect mediated by the tendency to deploy the simulation heuristic (Kahneman and Tversky, 1982)? That is, does belief in free will lead to punishment for intentionally-performed acts because high free will believers are more readily able to imagine a counterfactual outcome in which the actor refrained from performing the act?

#### A caveat

In this study participants completed the measure of free will beliefs after they had completed the critical independent and dependent variables. It is important to note that Clark et al. (2014) found that the desire to punish leads to greater endorsement of free will. Thus, it is plausible that participants' free will beliefs were driven *after the fact* by their reactions to the target. As such, one purpose of Study 4 was to examine whether the pattern would replicate when the free will belief independent variable *preceded* the moral judgment dependent variables. In addition, in Study 4 we turned again to the question of positive acts. Do free will beliefs influence perceptions of intent in prosocial acts just as they do for antisocial acts?

## Study 4

As noted, the literature on moral judgment has occasionally found positive/negative asymmetries; people often condemn immoral acts more extremely than they praise prosocial acts (e.g., Janoff-Bulman et al., 2009; Wiltermuth et al., 2010). Presumably this is because the presence of harm is perceived to be more harmful than the absence of benefits (Janoff-Bulman et al., 2009). Moreover, such findings are consistent with general loss aversion tendencies in human judgment and decision making (e.g., Kahneman and Tversky, 1979; Baumeister et al., 2001). Thus, there are grounds to believe that people might use the PIDI framework differently for positive vs. negative acts.

On the other hand, recall that the results across Studies 1–2 suggested that participants use proximal and distal intent similarly across blame judgments *and* praise judgments. Thus, we hypothesized that the pattern for praise judgments (Study 4) would parallel those of blame judgments (Study 3). Specifically, belief in free will would predict higher praise for the actor when the actor had proximal intent but not distal intent.

A second aim of Study 4 was to manipulate beliefs regarding free will/determinism. We reasoned that although there are reliable individual differences in belief in free will, both the free will position and the determinist position are intuitive to most people. Thus, compelling messages espousing either belief may override individuals' chronic belief. If the results with manipulated beliefs resembled the results with chronic beliefs, this would provide evidence that such beliefs play a causal role in shifting perceivers' emphasis toward proximal intent or distal intent.

## Method

### Participants

A total of 388 U.S. residents (70% female, mean age = 31.76, *SD* = 11.60, minimum = 18, maximum = 75) participated online via Amazon Mechanical Turk in exchange for $0.50. (Ethnic breakdown: 45% White, 19% East Asian, 14% South Asian, 3% Black, all remaining ethnicities <2%.) Sample size was determined with the effect sizes of Study 2 as a reference point. All participants provided informed consent.

### Procedure

To manipulate beliefs in free will vs. determinism, we used a procedure reported by Baumeister et al. (2009). The task was described as a “reading comprehension task.” Participants in the free will condition read a series of 15 statements (one per page, self-paced) that supported the idea of free will (e.g., “I have free will to control my actions and, ultimately, to control my destiny in life.”). Participants in the determinism condition read sentences supporting the idea of determinism (e.g., “Our actions are determined by what we have experienced in the past combined with the specific genetic predispositions that we have.”). After each sentence, the computer provided a text box into which participants were asked to type in the sentence they had just read. (This helped to bolster the cover story that the study was about short-term memory. It also served as an attention check).

Next, participants read one of four positive versions of the scenarios used in Study 1. In these scenarios, Alex does not wish to kill Linda. Instead, he wishes to save Linda's life. Distal Intent was varied by having Alex's thought be focused on the goal of saving Linda vs. the action of pulling a rope. Proximal Intent was varied by having Alex intentionally pull the rope to release Linda vs. accidently trip over the rope, causing it to release Linda. (See Supplementary Material).

Participants completed dependent measures that were analogous to those used in Study 1. Where appropriate, these items were reworded in terms of praise/positivity: (1) To what extent were Alex's actions intentional? (2) How much moral responsibility does Alex deserve for what happened? (3) How positively should Alex be judged? 4. How much praise should Alex receive as a result of his actions? (0–5 scales).

## Results and discussion

### Overall effects

Because the dependent measure items were highly correlated (Cronbach's α = 0.68), they were combined into a positive judgment index. Participants' scores on the index were submitted to a 2 (Distal Intent: present vs. absent) × 2 (Proximal Intent: present vs. absent) × 2 (belief manipulation: free will condition vs. determinism condition) ANOVA. Replicating the previous studies, this analysis revealed main effects for distal intent, *F*_(1, 380)_ = 179.95, *p* < 0.001, and for proximal intent, *F*_(1, 380)_ = 105.98, *p* < 0.001. The analysis also revealed a significant main effect for the belief manipulation, *F*_(1, 380)_ = 31.62, *p* < 0.001, indicating that those in the free will condition generally judged the actor more favorably than did those in the determinism condition.

### Effects involving manipulated free will/determinism beliefs

This analysis also revealed a belief manipulation × proximal intent interaction, *F*_(1, 380)_ = 6.29, *p* = 0.01. Neither the belief manipulation × distal intent interaction, *F*_(1, 380)_ = 0.01, nor the three-way interaction, *F*_(1, 380)_ = 0.42, were significant.

As in the previous studies, we attempted to pinpoint the effect of free will/determinist beliefs by testing within each scenario condition. Means are depicted in Table 4.

**Table 4:** **Study 4**.

	**Distal Intent**	
**Proximal Intent**	**Present**	**Absent**
**FREE WILL CONDITION**		
Present	3.74 (0.87)	2.42 (1.04)
Absent	2.46 (1.00)	1.35 (0.93)
**DETERMINIST CONDITION**		
Present	3.07 (0.76)	1.60 (0.78)
Absent	2.13 (0.97)	1.11 (0.79)

*Moral praise of the actor (0–5 scale) as a function of free will/determinist belief condition and the presence or absence of proximal intent and distal intent. (standard deviations in parentheses)*.

In the Proximal Intent Only condition, simple effects tests revealed that participants in the free will condition (*M* = 2.42, *SD* = 1.04) rated the actor more responsible than did those in the determinist condition (*M* = 1.60, *SD* = 0.78), *F*_(1, 380)_ = 19.82, *p* < 0.001, *d* = 0.90. In the Distal Intent Only condition, this difference was significantly reduced, *F*_(1, 380)_ = 3.36, *p* = 0.07, *d* = 0.34.

Simple effects tests also revealed that free will believers and determinists did not differ in the condition when both forms of intent were absent, *F*_(1, 380)_ = 1.72, *p* = 0.19, but did differ when both forms of intent were present, *F*_(1, 380)_ = 13.02, *p* < 0.001, *d* = 0.83.

In summary, the Study 4 data are consistent with Study 3 in that free will beliefs increased sensitivity specifically to proximal intent, not distal intent. This pattern held whether beliefs were measured (Study 3) or manipulated (Study 4). In addition, whereas previous research has associated belief in free will with higher punitiveness (e.g., Shariff et al., 2014), the present study suggests that this effect extends to praise for meritorious acts. Such a finding is consistent with recent results reported by Mackenzie et al. (2014) in which higher belief in free will predicted higher gratitude for a favor. Moreover, although the moral judgment literature has uncovered positive/negative asymmetries, in the present studies participants applied the PIDI framework equivalently to antisocial and prosocial acts. This suggests that people use the concepts of proximal and distal intent to calculate intentionality in general, not just for morally negative acts.

## General discussion

Most people assume that every action is preceded by an intention to act. Although there is considerable evidence that this assumption is flawed (e.g., Libet, 1985; Wegner et al., 2003), it remains a driving force in how both formal legal systems and laypeople reason about moral responsibility. But what exactly do people mean by “intention”? In the present studies, we (a) identified two important aspects laypeople's construal of intention and (b) identified two variables that predict preferential emphasis of one of those aspects.

In Study 1, when the actor had the malevolent goal in mind at the time of the act (even if he performed the act by accident), participants judged the distant actor more harshly than they judged the near actor. However, when the actor performed the act on purpose (even if he was not thinking about his malevolent goal), participants judged the near actor more harshly than they judged the distant actor. In Study 2, when the actor had the positive goal in mind at the time of the act (even if the he performed the act by accident), participants with a chronic tendency to identify actions at a high level judged the actor more favorably than those with a tendency to identify actions at a low level. In Study 3, when the actor did not have the malevolent goal in mind but did cause the death with awareness and control, those with a higher belief in free will rated the actor more responsible than did those with a lower belief in free will. In Study 4, a similar pattern was found when the actor performed a positive (life-saving) act and when free will/determinism beliefs were manipulated, rather than measured.

Thus, two well-studied, conceptually unrelated, social-psychological variables differentially predicted participants' sensitivity to proximal and distal intent. Taken together, these data suggest that the PIDI framework represents a useful way to organize observers' subtle but important shades of emphasis as they determine an actor's degree of intentionality and moral responsibility. It accounts for more variability than a binary distinction between “intentional” vs. “unintentional.” Consider, for example, that in Studies 1–2, construal level did not predict differential judgment in the clearly “intentional” condition (Both Present) or the clearly “unintentional” condition (Both Absent). However, construal level did predict more extreme judgment in one case of ambiguous intentionality (Distal Intent Only). In Studies 3–4, higher belief in free will predicted more extreme judgment in all conditions *except* Distal Intent Only. Thus, the PIDI framework allows researchers to specify with greater precision how a range of important psychological variables, including free will beliefs, psychological distance, and culture (see Plaks et al., manuscript under review) exert their effects on judgments of responsibility.

We wish to be clear, however, that we do not consider proximal and distal intent to be the sum total of ingredients of an intentional act. Indeed, numerous studies have focused on such elements as the actor's *belief* that their action will cause the outcome (e.g., Young et al., 2007), *desire* to cause the outcome (Cushman, 2008), *skill* to cause the outcome (Guglielmo and Malle, 2011), and *foreseeability of consequences* (Searle, 1983; Duff, 1990). The present studies build on such work by focusing specifically on two elements of intentionality that guide an action while it is occurring.

We view distal intent as a mechanism for translating a desire or plan into a specific act. When the actor has distal intent in mind while performing the act, she is mentally representing the specific end her act is addressing (i.e., “keeping her eye on the prize”). However, keeping one's eye on the prize is separable from focusing on the specific muscle movements necessary to perform the act. For example, during many complex actions (e.g., kicking a soccer ball into the goal), people may become temporarily so focused on “the prize” that attention to the specific motor action recedes. This corresponds to our Distal Intent Only condition. Similarly, during many complex actions, people may become temporarily so focused on the mechanics of task execution that they become “lost in the details.” At that moment, proximal intent may assume prominence over distal intent. This corresponds to our Proximal Intent Only condition.

A number of philosophers have found it useful to distinguish between these two aspects of intent (e.g., Goldman, 1970; Searle, 1983; Brand, 1984; Duff, 1990). The present work suggests that laypeople also make this distinction. These studies go on to highlight systematic intra- and inter-individual variation in points of emphasis within the generic model. Taken together, these data suggest that (a) the concepts of proximal and distal intent are intuitive to most people and (b) certain beliefs or foci will systematically elevate the prominence of one form of intent over the other.

### Implications for moral judgment

Prominent contemporary theories of moral judgment (e.g., Haidt, 2001; Greene, 2013) have focused on important questions regarding the role of emotion and reason in people's deontic vs. consequentialist tendencies. Perhaps because of the success of these approaches, considerably less research has focused on the role of intentionality in moral judgment. But there is much a focus on intentionality might add (see Gray et al., 2012; Malle et al., 2014). Consider that in typical scenarios presented to participants (e.g., the Trolley Problem), the focal act is committed with both proximal and distal intent present. What would happen, however, if we expanded the scenario to a full 2 × 2, PI × DI design? How might people's moral judgments differ if the actor pulled lever with only partial intentionally?

In a related vein, much of the literature in moral judgment has focused on the distinction between rational and intuitive/affective processes. Thus far, our approach has been silent regarding whether perceiving one type of intent relies more on rational, intuitive, or emotional processes. However, in future studies, one might, for example, introduce a cognitive load manipulation to the design to test whether any of the processes documented here are impaired. We suggest, however, that there is value to identifying the content of people's moral thought separately from modes of processing that in and of themselves possess no moral content. Doing so may allow researchers to identify important building blocks of moral thought that may, in turn, be used in different ways depending on whether the observer is using a more rational or more intuitive/affective mode of processing. (For similar ideas, see Malle et al., 2014).

### Implications for legal reasoning

The legal definition of many criminal offenses, including murder and rape, includes not only the act and its consequences (*actus reus*) but the concomitant thoughts, beliefs, and intentions of the actor (*mens rea*). Given that jury members are laypeople, there is a clear benefit to greater understanding of how laypeople reach their judgments of intent. The present studies identify beliefs and mindsets that orient people toward the ends or the means. Thus, a prosecuting attorney who wishes to have a jury focus on the actor's evil intent may do well to use language referring to the actor's thoughts, while the defense attorney may strategically use language that focuses on the action itself. Thus, it may be helpful for judges' instructions to alert jurors to such tactics.

Moreover, some theorists have argued that North American legal codes contain a bias toward “premeditation” (compared to judging the act itself) (e.g., 18 U.S.C. § 1111, 1999, cited in Malle and Nelson, 2003). Indeed, recent evidence suggests that North Americans place greater weight on distal intent than do East Asians and South Asians (Plaks et al., manuscript under review). Actions differ, however, in their degree of premeditation. Thus, North American legal codes may not be well-suited to handle cases of causal deviance, i.e., when the link between distal intent and proximal intent is disrupted (Denno, 2003; Malle and Nelson, 2003). For this reason, a fuller understanding of the components of intentional action may ultimately inform the writing of legal codes so that they reflect a broader palette of philosophical and folk-psychological concepts related to intentional action.

It is also important to note that the relationship in people's minds between responsibility and punishment is not a straightforward one (e.g., Cushman, 2008). People often mete out harsh punishments for largely unintentional acts (as in the case of “strict liability” crimes, such as statutory rape) and punish less harshly for some clearly intentional acts (e.g., killing in self-defense). The translation from moral responsibility to punishment is most likely influenced by a range of additional factors, including lay theories regarding the purpose of punishment (Carlsmith et al., 2002; Molden and Dweck, 2008).

### Future directions

Recently, researchers have suggested that intentionality calculations feature more prominently in observers' minds for some types of transgressions than for others (Young and Tsoi, 2013). For example, observers appear more inclined to emphasize outcomes over intentions for purity violations than for other types of violations (Young and Saxe, 2011). In future studies, researchers should investigate whether the PIDI model might, in fact, be less applicable when observers view the act as a purity violation rather than a fairness violation.

It is clear that proximal and distal intent are only two of numerous concepts related to the relationship between intent and moral responsibility. These include such legal concepts as “negligence,” “recklessness,” and “foreseeable consequences” and such psychological concepts as “blame” (Malle et al., 2014) and “culpable causation” (Alicke, 1992). In ongoing research, we are examining the relationships between proximal and distal intent and other components of intentional action in order to develop a more comprehensive model of lay theories of intentionality.

The Proximal Intent/Distal Intent framework is generative in that it implies numerous hypotheses for further research. For example, in an era of high profile, corporate financial malfeasance, how (if at all) does the pattern found in the present studies vary for white collar crimes? How (if at all) does the pattern vary for sins of omission compared to sins of commission? Do people show differential emphasis on proximal vs. distal intent when either the actor or the victim is a racial ingroup vs. outgroup member? Are the answers to any of these questions subject to cultural variation? These are topics of current research in our laboratory.

### Conflict of interest statement

The authors declare that the research was conducted in the absence of any commercial or financial relationships that could be construed as a potential conflict of interest.

## Footnotes

^1^The remaining questionnaires were: 1. Regulatory Mode Questionnaire (Kruglanski et al., 2000); 2. A self-esteem measure (Rosenberg, 1965); 3. My Religious Faith questionnaire (Cohen et al., 2003). These measures were included to test hypotheses that are not relevant to the present article.

^2^These measures were: demographics (age, gender, ethnicity); political orientation (one item); Regulatory Mode (Kruglanski et al., 2000); and Need for Closure (Webster and Kruglanski, 1994). These measures were included to test hypotheses that are not relevant to the present article.

^3^We also conducted analyses using the original four-item moral judgment index used in the previous studies. The results were equivalent those with the eleven-item measure. For example, the values for the proximal intent x free will beliefs interaction were: β = 0.13, t_(148)_ = 2.19, *p* = 0.03. Within the Proximal Intent Only condition, higher free will belief predicted harsher judgment: β = 0.45, *t*_(35)_ = 2.94, *p* = 0.006. The correlation between the four-item index and the eleven-item index was *r* = 0.93.
