# Supplementary material for: Construal level and free will beliefs shape perceptions of actors' proximal and distal intent
Source: Front Psychol. 2015 Jun 8;6:777. doi: 10.3389/fpsyg.2015.00777 (PMC4458567; doi:10.3389/fpsyg.2015.00777)
Supplement: Supplementary file 1 [file DataSheet1.DOCX]

APPENDIX A

Scenarios used in Study 1

**Both Present**

Alex wants to kill his ex-girlfriend Linda. He formulates a plan to push her into the lake during a camping trip they attend with the same group of friends every year. Knowing she can swim, Alex plans to tie her up with a rope before pushing her into the water. During their trip Alex convinces Linda to go for a boat ride with him. As they are about to get into the boat, docked in a secluded area by the lake, Alex takes a rope from the boat, ties Linda up and pushes her into the lake. Linda drowns.

**Distal Intent Only**

Alex wants to kill his ex-girlfriend Linda. He formulates a plan to push her into the lake during a camping trip they attend with the same group of friends every year. Knowing she can swim, Alex plans to tie her up with a rope before pushing her into the water. During their trip Alex convinces Linda to go for a boat ride with him. As they are about to get into the boat, docked in a secluded area by the lake, Alex takes a rope from the boat, and begins to tie her up. In trying to free herself, Linda actually tangles herself worse, falls off the dock into the water, and drowns.

**Proximal Intent Only**

Alex wants to kill his ex-girlfriend Linda. He formulates a plan to push her into the lake during a camping trip they attend with the same group of Linda every year. During their trip Alex convinces Linda to go for a boat ride with him. As they are about to get into the boat, Alex notices the safety rope is tangled. Thinking that he will later need the rope, he pulls it out so that they can untangle it. After thinking of the best method of untangling the rope, he directs Linda to hold onto one end and tries to untangle the rest by temporarily wrapping the rope around her. Unbeknownst to both of them, when Alex pulls on the rope it becomes wrapped a little too tight around Linda. It cuts off blood circulation so that she very quickly loses consciousness, falls into the lake and drowns.

**Both Absent**

Alex wants to kill his ex-girlfriend Linda. He formulates a plan to push her into the lake during a camping trip they attend with the same group of friends every year. During their trip Alex convinces Linda to go for a boat ride with him. As they are about to get into the boat, Alex notices the safety rope is tangled. He pulls it out so that they can untangle it. As they are untangling the rope, Linda trips, gets tangled in the rope, falls into the lake and drowns.

Scenarios used in Study 2

**All Scenarios**

Jane’s soccer team is tied with its arch-rival with one minute left in the championship game. Jane desperately wants her team to score a goal and win the championship. Jane has the ball at her feet near the opponent’s goal.

**Both Present**

Spotting an opening, Jane aims to shoot the ball toward the corner of the net. With precision and control, she kicks the ball right past the goalkeeper and into the corner of the net for a goal. Her team wins the game and the championship.

**Distal Intent Only**

Spotting an opening, Jane aims to shoot the ball toward the corner of the net. She kicks the ball, but it veers off course. However, it bounces off the leg of a defender and goes right past the goalkeeper and into the corner of the net for a goal. Her team wins the game and the championship.

**Proximal Intent Only**

Spotting an opening, Jane aims to pass the ball to a teammate who is standing between her and the goal. With precision and control, she kicks the ball right to her teammate. Her teammate is not expecting the pass and doesn’t see the ball zoom by her. However, the teammate is also blocking the goalkeeper’s view. The ball continues right past the goalkeeper and into the corner of the net for a goal. Her team wins the game and the championship.

**Both Absent**

Spotting an opening, Jane aims to pass the ball to a teammate who is standing between her and the goal. She kicks the ball, but it veers off course. However, it bounces off the leg of a defender and goes right past the goalkeeper into the corner of the net for a goal. Her team wins the championship and the game.

Scenarios used in Study 3

**Both Present**

Barbara wants to kill her husband, John. She formulates a plan to poison him while dining at a local restaurant. At one point during the meal, Barbara, thinking about killing John, slips some poison from the vial in her purse into John’s dish while he is away at the restroom. John returns and takes a bite of the now-poisoned food. The poison kills him virtually instantly.

**Distal Intent Only**

Barbara wants to kill her husband, John. She formulates a plan to poison him while dining at a local restaurant. At one point during the meal, Barbara, thinking about killing John, slips some poison from the vial in her purse into John’s dish while he is away at the restroom. John returns and takes a bite of the now-poisoned food. The poison is not strong enough to kill John. Instead, it makes the dish taste so bad that John changes his order. His

new dish contains a food that he is extremely allergic to. John eats the new dish and it kills him virtually instantly.

**Proximal Intent Only**

Barbara wants to kill her husband, John. She formulates a plan to poison him while dining at a local restaurant. At one point during the meal, John goes away to the restroom. Suddenly a spider crawls across John’s plate. Barbara hates spiders. Barbara, thinking only about killing the spider, takes the poison vial from her purse and drowns the spider on John’s plate. John returns and takes a bite of the now-poisoned food. The poison kills him virtually instantly.

**Both Absent**

Barbara wants to kill her husband, John. She formulates a plan to poison him while dining at a local restaurant. At one point during the meal, John goes away to the restroom. Suddenly a spider crawls across John’s plate. Barbara hates spiders. Barbara, thinking only about killing the spider, takes the poison vial from her purse and drowns the spider on John’s plate. John returns and takes a bite of the now-poisoned food. The poison is not strong enough to kill John. Instead, it makes the dish taste so bad that John changes his order. His new dish contains a food that he is extremely allergic to. John eats the new dish and it kills him virtually instantly.

Scenarios used in Study 4

**Both Present**

As part of their tradition, Alex and Linda go on a camping trip together. One afternoon they decide to go for a boat ride together. Linda walks ahead on the dock to get into the boat while Alex stops to light a cigarette before the ride. As Alex watches Linda get into the boat he sees her trip over a rope and get tangled as she falls into the lake. He decides to jump in a save her. He runs along the dock, jumps in and brings her to the surface just in time.

**Distal Intent Only**

As part of their tradition, Alex and Linda go on a camping trip together. One afternoon they decide to go for a boat ride. Linda walks ahead on the dock to get into the boat while Alex stops to light a cigarette before the ride. As Alex watches Linda get into the boat he sees her get foot get tangled on a rope, causing her to slip and fall into the lake. He decides to jump in and save her, but as he is running along the dock, he trips over the rope and hits his head on the side of the dock, incapacitating himself. Luckily, his trip causes the rope to come free from Linda’s leg and she is able to swim to safety.

**Proximal Intent Only**

As part of their tradition, Alex and Linda go on a camping trip together. One afternoon they decide to go for a boat ride. Linda arrives early to check the boating gear and notices that the safety rope is untangled. As she is trying to untangle it, the rope gets tangled around her and she slips and falls into the water. When Alex arrives at the dock he notices a rope dangling into the water. He begins to pull up the rope, not knowing that Linda is attached to the other end. His tug on the rope releases Linda and she swims to safety.

**Both Absent**

As part of their tradition, Alex and Linda go on a camping trip together. One afternoon they decide to go for a boat ride. Linda arrives early to check the boating gear and notices that the safety rope is untangled. As she is trying to untangle it, the rope gets tangled around her and she slips and falls into the water. When Alex arrives he notices the rope in the water, not knowing that Linda is attached to the other end. He trips over the rope and hits his head on the side of the dock, incapacitating himself. Luckily, his trip causes the rope to release from Linda and she is able to swim to safety.

APPENDIX B

Means and standard deviations for each of the moral judgment questions, *Study 1*.

Distance DI PI To what extent How much How negatively How much blame

were Alex’s responsibility does should Alex be should Alex receive

actions inten- Alex deserve? judged? for what happened?

tional?

near absent absent 3.47 (1.77) 3.53 (1.59) 3.94 (1.64) 3.76 (1.79)

present 4.11 (1.71) 4.94 (1.26) 4.72 (1.36) 4.89 (1.52)

present absent 4.43 (1.46) 4.19 (1.47) 4.13 (1.54) 4.13 (1.36)

present 5.82 (0.93) 5.69 (1.01) 5.31 (1.25) 5.71 (0.99)

far absent absent 3.32 (1.57) 3.53 (1.58) 4.21 (1.62) 3.58 (1.74)

present 3.33 (1.28) 4.19 (1.33) 4.19 (1.29) 4.52 (1.33)

present absent 5.74 (0.81) 5.68 (0.75) 5.56 (1.04) 5.74 (0.45)

present 5.68 (0.89) 5.77 (0.61) 5.72 (0.77) 5.58 (0.89)
